# A Pilot Study of a Sensor Enhanced Activity Management System for Promoting Home Rehabilitation Exercise Performed during the COVID-19 Pandemic: Therapist Experience, Reimbursement, and Recommendations for Implementation

**DOI:** 10.3390/ijerph181910186

**Published:** 2021-09-28

**Authors:** Veronica A. Swanson, Vicky Chan, Betsaida Cruz-Coble, Celeste M. Alcantara, Douglas Scott, Mike Jones, Daniel K. Zondervan, Naveen Khan, Jan Ichimura, David J. Reinkensmeyer

**Affiliations:** 1 Department of Mechanical and Aerospace Engineering, Henry Samueli School of Engineering, University of California, Irvine, CA 92697, USA; swansonv@uci.edu; 2 Department of Outpatient Physical Therapy, University of California, Irvine, CA 92868, USA; vchan2@uci.edu (V.C.); bcruzcob@uci.edu (B.C.-C.); celesa1@uci.edu (C.M.A.); 3 Division of Rehabilitative Services, University of California, Irvine, CA 92868, USA; dscott1@uci.edu; 4 Virginia C. Crawford Research Institute, Shepherd Center, Atlanta, GA 30309, USA; mike_jones@shepherd.org; 5 Flint Rehabilitation Devices, LLC, Irvine, CA 92614, USA; dzondervan@flintrehab.com; 6 Pt Pal, Cherry Hill, NJ 08003, USA; naveen@ptpal.com; 7 Department of Physical Therapy, Acute Rehabilitation Unit, University of California, Irvine, CA 92868, USA; jichimur@uci.edu; 8 Department of Anatomy and Neurobiology, UC Irvine School of Medicine, University of California, Irvine, CA 92697, USA

**Keywords:** physical rehabilitation, mRehab, telerehabilitation, implementation, COVID-19, mobile applications, remote physiologic monitoring, RPM, HEP, home exercise program

## Abstract

Adherence to home exercise programs (HEPs) during physical rehabilitation is usually unmonitored and is thought to be low from self-reports. This article describes exploratory implementation of a Sensor Enhanced Activity Management (SEAM) system that combines HEP management software with a movement sensor for monitoring and motivating HEP adherence. The article also presents results from attempting to gain reimbursement for home use of the system with therapist oversight using Remote Physiologic Monitoring (RPM) codes. Four therapists used the system in their regular practice during the first six months of the COVID-19 pandemic. Therapists filled out surveys, kept notes, and participated in interviews. Billing and reimbursement data were obtained from the treatment facility. Exercise data from the SEAM system were used to understand HEP adherence. Patients were active for a mean of 40% (26% SD) of prescribed days and completed a mean of 25% (25% SD) of prescribed exercises. The therapists billed 23 RPM codes (USD 2353), and payers reimbursed eight of those instances (USD 649.21). The therapists reported that remote monitoring and the use of a physical movement sensor was motivating to their patients and increased adherence. Sustained technical support for therapists will likely improve implementation of new remote monitoring and treatment systems. RPM codes may enable reimbursement for review and program management activities, but, despite COVID-19 CMS waivers, organizations may have more success if these services are billed under supervision of a physician.

## 1. Introduction

Successful outcomes for physical rehabilitation programs depend on patients’ completion of therapeutic exercises prescribed to be completed outside of the clinic [1,2]. However up to 65% of patients are nonadherent or only partially adherent to their home exercise programs (HEPs) [2], with estimates varying across studies [1,3]. Patient diaries and self-reported questionnaires are largely used to measure adherence, making it difficult to obtain accurate information [2,4]. A comparison of patient-completed exercise diaries and data gathered from concealed sensors in exercise equipment found that patients over reported their activity by 25% on average [5]. In a study of patient adherence to exercise programs for chronic low back pain (*n* = 61), 39% self-reported as being completely adherent, but therapists perceived that only 16% of participants were completely adherent, and only 15% of participants were able to recall all of the exercises contained within their program and demonstrate them accurately [6]. In a survey delivered to physical therapists, only 36% of therapists reported high levels of adherence to home exercises among their patients. Among the factors reported to affect adherence, 81% of therapists responded that forgetting to do their exercises was a barrier for their patients, and 64% of therapists reported that patients forgot how to do their exercises. Eighty-two percent of the physical therapists that responded provided verbal education about patients’ HEPs, and of those therapists, 45% used verbal instructions exclusively [7].

Nonadherence is a multidimensional process not easily solved including internal factors (such as patient’s locus of control, depression, belief in importance of activities) and external factors (such as supportive environment and access to transportation) [1,6]. Digital delivery of HEPs has already been shown to enhance patient adherence [8,9,10,11] and with the relatively high rate of smartphone ownership [12] there is a growing body of work investigating the use of smartphones, apps, and sensing technologies to facilitate rehabilitation and remote care [13,14,15,16]. However there has been limited study of implementation of mobile health applications in routine care settings [13,16], and most efforts have focused on specific medical conditions [15] or have a limited set of exercises which can be performed using the system [16,17,18]. We therefore aim to contribute exploratory findings from a clinical setting of a system designed for general use with narratives from participating therapists and adherence data from patients.

This preliminary study was initiated in response to the COVID-19 pandemic. Rehabilitation is an essential service for patients [19,20,21,22] that needs to be continued during the COVID-19 pandemic [23]. However, the practice of physical rehabilitation itself can pose a risk of exposure to the virus for patients and healthcare practitioners alike, and investigation is needed for methods to continue provision of care while mitigating risks. If adherence was problematic before the pandemic, the issue has likely been further compounded with reduced face-to-face contract and group organized rehabilitation. Telerehabilitation services may provide a partial solution [24,25]. What is learned and developed now during the pandemic can potentially provide a basis for expanding use of telerehabilitation in the future.

The goal of this study was to investigate the feasibility and implementation of a novel Sensor-Enhanced Activity Management (SEAM) system [26] for physical rehabilitation and explore its use during the COVID-19 crisis. This effort is part of the Rehabilitation Engineering Research Center for Mobile Rehabilitation (mRehab) RERC, the purpose of which is to “ensure ICT (Information and Communication Technology) access by people with disabilities in order to advance the development and use of mobile rehabilitation technologies that will improve adherence, engagement, and outcomes of home- based therapeutic interventions” [27]. The interest in applying mobile technologies to adherence with HEPs was motivated by survey work investigating use cases for mRehab in which 73.9% of physical therapists who responded identified supporting patient adherence to prescribed exercises and activities as a desired use case [28].

The pilot investigation of reimbursement feasibility was made possible because of expanded guidelines for Remote Physiologic Monitoring, sometimes referred to as Remote Patient Monitoring, (RPM) codes (see Table 1) set by the Centers for Medicare and Medicaid Services (CMS) during the COVID-19 pandemic. Specifically, CMS expanded the type of healthcare professionals who were allowed to furnish and bill for remote monitoring services to include physical therapists and occupational therapists, making it possible to implement a remote monitoring and treatment system that might provide return on investment for a physical therapy clinic [29]. CMS first introduced a separately payable RPM code in 2018, and then created three new RPM codes (99453, 99454, 99457) in 2019 to more accurately reflect how RPM services are furnished using current technology and staffing models [30,31]. Finally in 2020, CMS added a fourth RPM code, 99458, which allowed for additional monitoring time (past the first 20 min of service billed with 99457) to be billed in a single month. At this time, it was clarified that RPM services could be furnished “incident to” under general supervision of a physician [32]. Further details on the use of RPM codes can be found in Text S1. There is limited existing research on the use of RPM codes [33].

**Figure 1:**
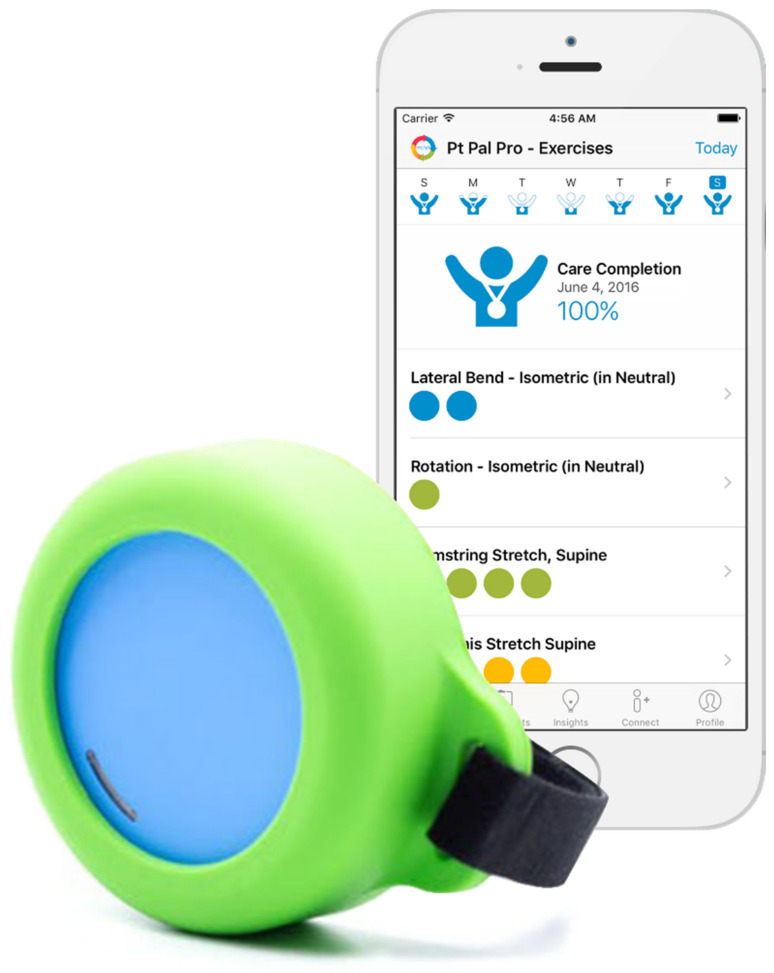
FitMi sensorized puck (blue) and silicone–Velcro strap (green) and Pt Pal patient app. The puck can be held and moved, squeezed, placed on a table or the floor and pressed, or strapped to a limb.

The SEAM system we investigated is the combination of two existing commercial products: Pt Pal [34] and FitMi [35] (Figure 1).

Pt Pal is a cloud-based clinical patient engagement platform that assists healthcare providers in managing patient care via patients’ mobile devices and web-based portals. FitMi is an interactive exercise tool designed for stroke rehabilitation that interfaces two puck-like sensors with software that guides users through 40 therapeutic, gamified, exercises, interactively recording and responding to movement activity via an embedded accelerometer, gyroscope, and load cell. Through the integration of these two products, the SEAM system offers a method of remotely delivering HEPs for physical therapy and collecting objective data about patient adherence. Through a web interface, therapists can use Pt Pal to prescribe HEPs choosing from a large library of exercises, monitor patient adherence, and manage HEP progression. Patients use the Pt Pal app to access and complete exercises. The app tracks patient initiation of exercises and the amount of time the patient interacted with the exercise up to completing the full prescribed exercise time. The FitMi pucks can be used during exercises to track activity accomplished during exercise. Thus, we designed the SEAM system to combine the benefits of a robust HEP management and patient engagement platform in Pt Pal with objective movement data from the FitMi pucks to assess adherence with prescribed exercises.

The aims of this pilot study were the following: to investigate usefulness of a remote patient monitoring system during the COVID-19 pandemic from the perspective of rehabilitation therapists, in particular with respect to objective compliance data; to identify areas of improvement in the integration of these two existing systems; to investigate important aspects of implementation as identified by treating therapists and study team members; and to test the use of remote patient monitoring codes for reimbursement.

## 2. Materials and Methods

Participants: Three therapists from outpatient services of University of California, Irvine Medical Center participated in the pilot study, two Physical Therapists (PT) and one Occupational Therapist (OT). UCI Medical Center is situated in an urban setting and has 411 licensed beds. It is the principal clinical facility for the teaching and research programs of the UC Irvine School of Medicine and is designated as Orange County’s only Level I Trauma Center.

This study focused on implementation issues experienced by therapists using existing healthcare technologies to perform their normal treatment activities, and we did not acquire protected health information. Therefore, this study was determined exempt from full review following the UCI IRB’s exempt self-determination tool. The study was confirmed by the UCI IRB, participants provided informed consent, and study staff followed all relevant Human Research Protection Program policies and procedures.

### 2.1. Study Design

Therapists were provided two initial training sessions by the product development teams, Pt Pal and Flint Rehab: an initial 3.5 h training session followed by a 2 h training session two weeks later. The first session consisted of an overview of the study by the study team, an overview of the technical system by the product development team, a hands-on walkthrough of several key features, and a demonstration of the typical set-up process for a patient. Therapists were instructed to explore the system and practice in between sessions. On returning for the second session, the study team addressed questions from the therapists and reviewed the set-up procedure. Therapists were given user manuals for reference and encouraged to reach out to the study team if they had questions or technical difficulties. The study team provided on-site troubleshooting and offered assistance, adding exercises to the system if the therapists chose to. Training on the RPM billing codes was also delivered in the initial training sessions given by the study team. Therapists’ time spent on study-related activities (training, practice, documentation of study activity) was funded by the study.

Patients were invited to use the technology according to their therapists’ judgement of candidates’ suitability across factors such as diagnosis, cognition, patient interest, and patient acceptance of the technology. No restrictions were made on patient diagnoses or demographics and no mandates were given on the types of activities that should be performed with the patients. The only restriction was that participants must have a smartphone, and we asked the therapists to enroll patients with a variety of health insurance payer sources if possible. Treatment decisions such as duration of care or specific rehabilitation activities were left to the discretion of the treating therapist. Therapists were encouraged to use videoconferencing technologies such as Zoom to conduct treatment sessions if they felt it was appropriate.

### 2.2. System Description

The SEAM system integrates two existing systems: Pt Pal and FitMi. Pt Pal provides a web-based portal that allows therapists to prescribe exercises to patients, which are then viewed on the patient’s phone in an app. There is an extensive library of existing exercises or therapists can choose to upload their own exercises. The library of existing exercises included 40 gamified exercises designed explicitly for use with the FitMi pucks, which were based on the RehabStudio PC software that normally comes with FitMi. Patients open the app and connect to their FitMi puck via Bluetooth. In the app, they are presented with a list of exercises that their therapist has prescribed for them on that day. When patients select an exercise by pressing the exercise icon, they are first presented with written instructions describing how to perform the exercise. Exercises also include a picture of the exercise or a video showing the exercise being performed. This video can be a link to an existing video, or a recording made using the patient’s phone. Patients then tap a button presented on the app screen to begin the exercise which (depending on the style of exercise prescribed) will play a visual and auditory sequence cuing when patients should perform movement repetitions and when they should rest. While performing the exercise, patients hold the FitMi puck as they move, or squeeze the puck, or strap the puck to their leg or arm as directed by their therapist for each exercise. The accelerometer and gyroscope within the puck are used to count distinct movements of the puck and the force sensor detects when the puck is pressed or squeezed during the activity. The web-portal records if the patient initiates the exercise, completes the exercise, and the number of movements or squeezes experienced by the puck during that exercise. If a patient stops an exercise early, or the therapist has indicated they should receive this survey, they are prompted with a feedback survey asking about the patient’s pain and difficulty performing the activity. Feedback is reported numerically in a table format as well as graphically in the web-portal and can be used to help track a patient’s experience over time. Secure messaging within the app enables patients and therapists to communicate as desired.

### 2.3. Data Collection and Statistical Analysis

Several data sources were used. Therapists filled out surveys after technology training sessions and after patient treatment sessions. The Post Therapy Session survey focused on perceived ease of using the system with their patient and their perception of their patient’s satisfaction with the SEAM system. Patients were not asked to fill out a survey about their satisfaction during treatment sessions to avoid additional time burdens on treatment sessions. Paired sample, two-tailed *t*-tests were used to compare therapist’s perceived ease of use and perceived patient satisfaction on the first day of use with a patient and the last recorded day of use with that patient. Each therapist kept a diary to record observations or notes that they felt were not captured by the surveys, and therapists kept logs tracking time spent towards study-specific activities (training, practice, documentation) and tracking the RPM codes billed for SEAM use.

The study team also conducted mid-study discussions with therapists to collect information and answer questions, and end-of-study interviews with each therapist. End-of-study interviews were recorded, transcribed using Otter.ai, and checked against recordings for correctness. Transcripts of interviews with therapists in the outpatient setting were qualitatively analyzed using summative content analysis. Qualitative analysis codes were inductively generated. Frequency of codes was used to highlight repeated comments and identify larger themes related to therapists’ experiences and guide understanding of relative importance between codes and themes. Discussions with members of the management teams and the inpatient therapist were used to inform material presented in the discussion.

Finally, therapists administered discharge surveys to participating patients when they were discharged from their care. This survey contained items from the Intrinsic Motivational Inventory (IMI) [36,37], questions related to the patient’s experience with the SEAM system, and questions to the therapist regarding their experience with the SEAM system and this particular patient. The survey contained four IMI categories: Value or Usefulness, Interest or Enjoyment, Effort or Importance, and Perceived Competence. Surveys used in this study can be found in Text S2.

## 3. Results

After the initial training session, the therapists rated how comfortable they felt using the SEAM system on a scale from 1 (Very Uncomfortable) to 5 (Very Comfortable; see Table 2).

Therapists 1 and 2 gave lower self-ratings after the initial training than Therapist 3 and subsequently spent more time on training activities outside of the sessions delivered by the study team. Therapist 3 later reported they would like more practice activities to be built into the training sessions, as they personally prefer structured to unstructured training styles. Only one therapist requested assistance creating new exercises to include in PTPal. To aid in this, a member of the study team reformatted images the therapist had such that they met the dimension requirements of the system. Ten patient participants were recruited (Table 3). One patient, SEAM 02, was enrolled but dropped out after discovering their insurance would not pay for the total of their treatment costs. One patient, SEAM 06, was enrolled in the inpatient setting where RPM codes could not be billed. These patients are not included in the results.

### 3.1. Adherence

Deidentified Pt Pal activity records were analyzed to summarize prescriptions patients were given (Table 4) and quantify their adherence (Table 5).

SEAM 04 self-discharged from care early and had minimal interaction with the system. On average, patients were active for M = 40 (26 SD) percent of prescribed days of exercise. Patients completed M = 25 (25 SD) percent of their prescribed exercises. Of the exercises that patients attempted (i.e., started and completed or started and ended before prescribed duration), M = 20 (27 SD) percent of these exercises had associated device data reported as the number of movements or squeezes captured by the puck sensors during the exercise.

### 3.2. Survey Responses

From therapists’ responses to the post therapy session surveys, on average, therapists rated their patients’ initial satisfaction (1 Very Dissatisfied, 5 Very Satisfied) as M = 3.9 (0.69 SD) and final satisfaction (or their last reported value) with a rating of M = 4 (1 SD). Therapists rated their initial ease of use in using the system with a patient during a visit (1 Very Difficult, 5 Very Easy) as M = 3.3 (0.49 SD) and their final (or their last reported value) ease of use with a patient as M = 4.1 (0.69 SD). The change in patient satisfaction rating between the start and end of treatment for all patients was not significant (*t*-test, *p* > 0.05); however, the change in therapists’ reported ease of use between start and end of treatment for patients was significant (*t*-test, *p* < 0.05).

At the time of writing, four of eight patients were discharged from care, and three of the four patients were administered the discharge survey by their therapists. On a 1–7 scale, with 7 showing agreement, their average response is as follow: value or usefulness M = 6.33 (0.58 SD), interest or enjoyment M = 6.50 (0.50 SD), effort or importance M = 6.67 (0.58 SD), and perceived competence M = 5.17 (1.61 SD). Thus, these three patients found working with the SEAM system interesting, enjoyable, and of importance to them.

In open-ended questions, patients expressed appreciation for the structure and progress the system provided, and patients noted that the videos of exercises embedded in the system were particularly helpful. Patients expressed dissatisfaction with the physical movement sensor used to capture data, primarily because of its bulkiness (Table 6). All three responding patients expressed they were “Likely” or “Very Likely” to continue using the system after discharge. SEAM 05 and SEAM 07 participated in some telerehabilitation using video conferencing. These patients agreed that the SEAM system made telerehabilitation easier to perform. When asked if using the SEAM system made them more comfortable getting physical therapy during the COVID19 pandemic, SEAM 05 responded “Strongly Agree,” SEAM 07 responded “Agree.” Therapists noted that the ability to embed videos of prescribed exercises into the app was useful and that the system was useful for promoting motivation and adherence. On average, therapists responded that the system was moderately easy to use with their patient M = 3.25 (0.5 SD) (1—very difficult, 5—very easy), and their patients appeared satisfied with the SEAM system, M = 4.25 (0.96 SD) (1—very dissatisfied, 5—very satisfied).

### 3.3. End of Study Interviews

Summative content analysis was used to codify end-of-study interviews to distill repeated comments and identify larger themes related to their experiences using the system during the study period. A summary of this analysis is shown in Table 7. Response concepts coded were organized by positive or negative sentiment, and then further organized into thematic categories and subcategories. Positive themes related to the value the system provided to patients, and ways in which the system facilitated providing care to patients. Negative themes were stratified into themes related to implementation, potential system improvements identified by therapists, and technical issues. The number of mentions of each code by each therapist is given to highlight the diversity of experiences and opinions. Though there were 30 different codes identified in this analysis, 6 codes (3 positive and 3 negative) were mentioned frequently enough that they each represented 5% or more of the total distinct mentions found in this analysis (*n* = 104). These highlighted codes were adherence, patient satisfaction, and patient motivation within the positive sentiment codes, and patient attributes, pucks not recording/performing as expected, and Bluetooth problems within the negative sentiment codes.

### 3.4. Billing and Reimbursement

The therapists billed 23 RPM codes for a total of USD 2353 and payers reimbursed 8 of those codes for a total of USD 649.21 (see Table S1 for full reimbursement data). The main items that were denied were codes 99457 and 99458, which are for the first 20 min of remote physiologic monitoring performed in a month and an additional 20 min of services furnished in that same calendar month. Of the 23 codes billed, 14 of them were for 99,457 and 99458, and 85% of those charges were denied, primarily because payer sources reported they were not covered (see Table 8). However, both Cigna and the United Healthcare Medicare Managed Care plan denied one unit of 99457 and reimbursed one unit of 99457. Blue Cross denied all the RPM codes billed but cited that these patients’ contracts for PT had already reached the maximum reimbursable amount for a given day before these RPM services were billed.

**Figure 2:**
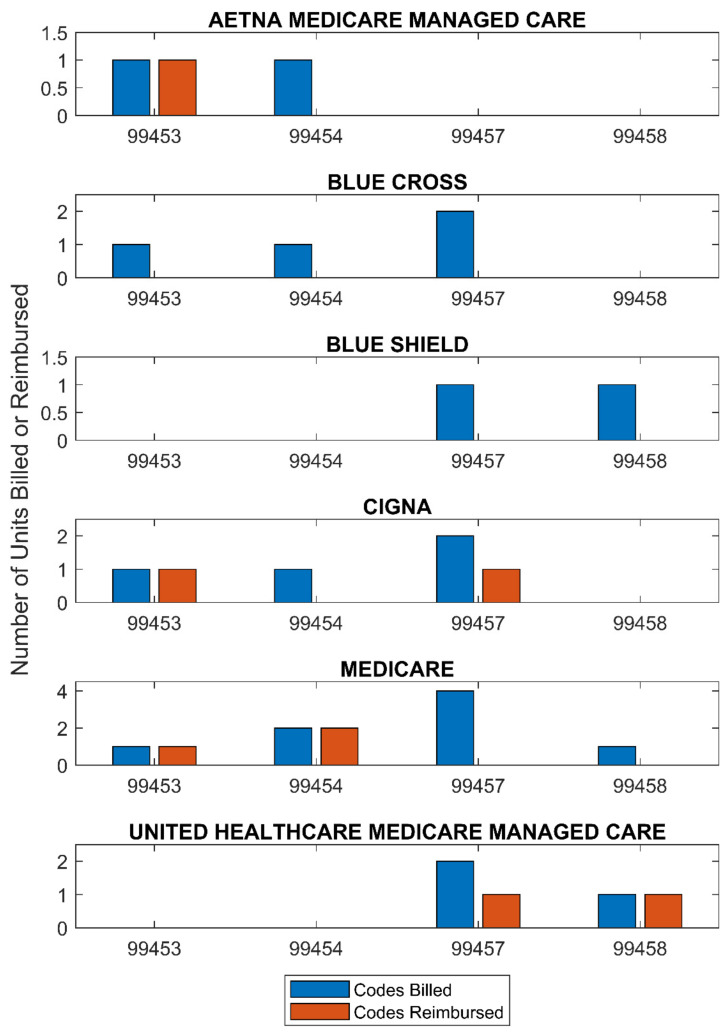
Chart of codes billed (blue) and codes reimbursed (orange) organized by payer.

Several codes that were eligible to be billed were missed as seen in the missing 99453 and 99454 values under Blue Shield and the United Healthcare Medicare Managed Care rows of Figure 2. Billing opportunities were missed due to confusion related to how to use the codes and forgetting to bill as part of a new workflow.

SEAM 05’s insurance, Blue Cross, rejected the RPM billing codes. These codes were not listed as approved under physical therapy in their insurance policy, however the codes the facility regularly used for in-person therapy were approved. As such, the care team only billed SEAM 05’s insurance for in-person therapy.

## 4. Discussion

We developed and tested a Sensor Enhanced Activity Management (SEAM) system that combines HEP management software with a movement sensor. The SEAM system remotely captured objective data regarding HEP adherence, demonstrating that patients’ adherence was partial (patients completed 25% of prescribed exercises on average). Nonetheless, therapists expressed the opinion that remote monitoring with the physical movement sensor was motivating to their patients and appeared to improve patient adherence compared to their experience with conventional HEPs. Reimbursement was partial as well, with 8 of 23 codes being reimbursed, but therapists still expressed the desirability of even partial reimbursement. We now discuss these findings, elaborating on them by sharing brief quotes obtained during interviews with the study therapists. We will focus first on the results concerning adherence and motivation, then on our findings on reimbursement for remote patient monitoring, then conclude with study limitations and recommendations for future research.

### 4.1. Adherence and Motivation

In our coding analysis of the therapists’ comments, codes related to adherence/accountability, patient motivation, and patient satisfaction were the most frequently mentioned codes that expressed positive sentiment. Therapists felt that the addition of a physical device with which to perform their exercises was beneficial to patients because it conveyed to patients that their therapist was monitoring their activity. Therapists conveyed these sentiments in interview quotes such as these:

Therapist 1: “*One thing I learned was that the use of the technology is highly motivating. Initially my patient was being non-compliant, and after the introduction of this technology they were doing their homework and going for walks*.”

Therapist 2: “*It really helped keep my patients on task with their home exercise program. Because they knew that they were being monitored, I think that helps them to be more compliant with their home exercises*.”

Therapist 3: “*Very beneficial, even just having the puck there, believe it or not. I felt just having that there, even if it wasn’t necessarily capturing all of their repetitions, it was just something that reminded them ‘this is something that I’m doing.’ Versus just sitting there and you’re stretching your hand or your arm, here you’re given something that’s visual or tangible that helps keep you on track*.”

Therapist 3: “*Overall, it did help compliance and accountability. When they walked in, if they didn’t do their exercises that week, they already came in with the “I’m so sorry, this is the reason why I didn’t get it done.” For me, I felt like it was always in there, in the back of their mind. They knew I had a way of seeing that they did or did not complete their exercises*.”

What is clear from these quotes is that the therapists viewed the incorporation of sensors into home exercise programs in a positive light, particularly insofar as the sensors served as a motivational aid to their patients. This is consistent with existing literature examining the effect on exercise adherence of remote monitoring programs [9,11]. The primary body of literature discussing home exercise literature for rehabilitation reports adherence by percent of participants that report complete adherence to their prescribed programs. Here we find that none of our participants were completely adherent (Table 5), but the system produced objective data on participation at the level of the specific exercise prescribed. Such methods will facilitate further study on patient adherence and potential factors which influence it.

### 4.2. Reimbursement for Remote Patient Monitoring

Therapists also appreciated that use of the SEAM system allowed them to at least partially bill for services similar to what they were already providing, but that they could not previously bill for. As an example, consider the following quote:

Therapist 2: “*Having the ability to bill the insurance for some remote patient monitoring really helped make my time more productive, from a billing perspective. When the pandemic first started, and the clinic was closed, I was making a lot of phone calls to patients and asking them questions and giving them advice, but I wasn’t able to bill for any of that. But with this system, that could be very useful*.”

However, some billing attempts were not reimbursed. We identified three main reasons. First, confusion related to the billing codes may also have led to missed billing opportunities. Therapist 2 stated that they thought they may have underbilled for their services at the start of the study, but that they became more confident in the use of the codes as the study progressed. Therapist 3 stated that at one point during the study they billed a code out of order. This was corrected after a member of the facility’s billing department reached out to them. Evidence of missed codes can be seen in the full reimbursement table (Table S1) and Figure 2. SEAM 07, SEAM 08, and SEAM 09 have codes 99457 and 99458 codes billed, but not 99453 or 99454, which are meant to be billed at the start of service. Missed billing opportunities further emphasize the need for investment in initial training.

Second, Blue Cross denied all the RPM codes billed during this study. They reported that these patients’ contracts for PT had already reached the maximum reimbursable amount for a given day before these RPM services were billed. Third, most of the 99457 and 99458 codes billed were denied and payers stated that these codes were not covered. Billing for these codes appear to have been rejected because of confusion related to who can bill for and furnish RPM services. SEAM 05′s insurance denied the claim, specifically citing that pre-authorization from a physician was required.

The current CMS clarifications for 2021 state that RPM codes are listed under Evaluation and Management (E/M), and therefore can only be billed by physicians or NPPs who are eligible to bill Medicare for E/M services [38], where an NPP is defined as a non-physician practitioner, defined separately from a qualified therapist such as a PT or an OT [39].

However, we note that the COVID-19 Emergency Declaration Blanket Waivers for Health Care Providers states:

“*This allows health care professionals who were previously ineligible to furnish and bill for Medicare telehealth services, including physical therapists, occupational therapists, speech language pathologists, and others, to receive payment for Medicare telehealth services*” [29].

Furthermore, the declaration stated that the blanket waivers are in effect, with a retroactive effective date of 1 March 2020, through the end of the emergency declaration, which at the time of writing, has not ended. Following these clarifications, it is the opinion of the research team that RPM services should have been reimbursed under the COVID-19 waiver. For further explanations of billing RPM codes, as well as Chronic Care Management Codes, in the context of Medicare, the reader is referred to another recent paper [33], which presents the design of a “Safe at Home” program and includes theoretical projections for how it can be reimbursed.

### 4.3. Limitations, Recommendations, and Future Directions

The most consistently cited technical issue during this pilot study was that repetitions captured by the sensors did not quantify patient activity with enough accuracy. Developing robust algorithms for accurately counting exercise repetitions or engagement is an important direction for future research.

Another important direction is developing sensor-based methods to discern whether exercises are done appropriately. The system demonstrates how to do the exercises accurately through pictures and written instructions, and, for some exercises, through videos and verbal instructions that are played during the exercise (such as “begin” and “rest”). Therapists also rehearsed the exercises with patients in-person. However, a current limitation is that the sensor system does not detect the accuracy of exercise completion. Judgment of movement quality is a problem with the current standard of care as well, which is paper-based exercise. Availability of sensors may help in providing movement quality information. For example, we found that a measurement of movement smoothness obtained with a wrist-worn sensor encoded information about movement performance separate from that carried by standard clinical scales, and was potentially related to movement quality [40]. Movement smoothness also correlates with patients’ self-report of movement quality using established clinical scales [41].

Patients found the sensor puck was bulky to wear on a limb and difficult to grasp if they had limited hand function or strength. We are investigating using a smaller “clip-like” sensor in response to this finding. Finally, therapists and patients also reported issues connecting to the pucks from the app via Bluetooth. In response to these reported issues, the development team has redesigned the system workflow such that the app prompts the user to initiate the Bluetooth connection when applicable and will also notify users if the relevant settings are not enabled, reducing the number of steps a patient must initiate on their own.

We did not track patient eligibility in this study, but, given their experience with the system, we asked therapists to estimate the percent of their patient population that could be eligible to use the system and would benefit from the system (Table 9).

Therapist 1 reviewed their current case load and estimated the smallest eligibility citing barriers of technological fluency, lack of a smart phone, and diagnosis related complications were cited as potential barriers. Therapist 3 reported that all of their patients would likely benefit from the portability and accountability provided by the system even if their patients experienced difficulties with the system. Clearly, there is substantially variability in therapists’ estimates of eligibility, in part due to the specific features of the patient populations they treat. Future studies should rigorously track eligibility and patient experience with such technology for different patient populations.

As suggested by therapists in this study, future directions of study for the SEAM system could involve the use of the remote monitoring and treatment system to provide HEPs to patients between gaps of care. Depending on demand and capacity, patients can wait for months between discharge from an inpatient facility and evaluation at an outpatient facility and wait further time still between their evaluation at the outpatient facility and the start of their treatment. Whether it will be possible to bill for such a service during gaps of care is unclear, yet the therapists we interviewed believed it would be an effective strategy to prevent patient health decline and loss of motivation.

Another important direction for future research is to study how patterns of HEP prescriptions, such as number of exercises or types of exercises, influence patient adherence [42]. Incorporation of systems like SEAM into routine practice will make this sort of analysis possible using large scale data. This in turn will usher in a new era of evidence-based guidelines for optimizing HEP prescriptions.

Finally, in the authors’ opinion, training with the therapist care team is crucial for implementing new technologies such as SEAM. Therapists are the access point to the system for patients, and if they are not comfortable with the system, they will use alternative methods with which they are more familiar [28,43]. However, working against sufficient training is the fact that therapists have little time in the clinic outside of patient visits and some allotted documentation time [44]:

Therapist 3: “*Overall, the biggest challenge is time. And it’s not the study or the program, it’s just time. Being able to incorporate something new and novel into your program takes a little bit more time and effort to be proficient with it. And with some of the constraints that we have, you’re always pressed. I think it’s just the nature of the beast of how healthcare is right now. Everything is just maximize time, maximize productivity, and then everything else kind of falls in wherever it can*.”

Troubleshooting the system, navigating the software interface, and creating exercise programs for patients were the most cited time concerns.

To minimize these time demands, we suggest three approaches. First, we drafted a recommended training schedule that builds on the training delivered at the start of this study (see Text S3). The schedule recognizes initial training sessions alone are not sufficient, because not all issues can be foreseen, since they vary by therapist and patient.

Second, we suggest that it will be helpful to have a technical assistant to assist with troubleshooting patient setups and perform initial system installation for patients. Such an assistant could have “office hours” or a technical assistance hotline for patients to contact to preserve treatment time for therapy and relieve therapists from the role of troubleshooting. An assistant in this role could be a member of the facility’s IT support staff, or, perhaps, a volunteer who has learned to use the technology.

Third, although the system is designed to be used with a wide variety of patient types and diagnoses, careful selection of initial patients may increase therapists’ success when they are new to the system. Indeed, patient attributes were one of the most frequently mentioned codes in our coding analysis, as exemplified by the following quote:

Therapist 3: “*There are definitely diagnoses that I feel it would work really well with, higher level patients that basically you give your home exercise program to, you’re able to monitor it, you check in with them a couple times a week, just to make sure that they’re doing it safely, that they’re doing them correctly, or if they have any questions. Some of the other patients, with more moderate to severe impairments, it’s definitely challenging because, one of the big things that we do as therapists is putting our hands on the patients to help facilitate movements or inhibit movements and you just can’t do that via telerehab*.”

Therapist 1 enrolled only a single patient during the study due to the complexity of their patients’ conditions. Selecting patients with intact cognition, available caregiver support, and at least moderate technology fluency will facilitate implementation.

This work represents preliminary investigation of feasibility and implementation of a remote patient monitoring system in an outpatient physical therapy setting and provided unique insight under the conditions of the COVID-19 pandemic. To our knowledge, existing literature on the use and implementation of RPM codes is quite small and this is the first study performed in the domain of physical rehabilitation. The numbers of therapists and patients studied were small. In addition, the focus was on therapists’ experience, and future work should analyze the user experience from the patient perspective in a larger population of patients. Future studies should also closely track patient eligibility statistics as well as formal clinical outcomes achieved by using the system.

However, despite these limitations, the study provides a proof of concept that can inform future work. For example, the observations and findings presented here should help inform designers of mRehab interventions for clinical and home use about therapist perspectives and some of the relevant contextual and implementation considerations. The study team will use the findings to inform future implementation work following improvements to the system and a subsequent multi-site efficacy randomized controlled trial.

## 5. Conclusions

SEAM provided a remote monitoring platform for therapists to provide treatment remotely or in-person. RPM codes allowed therapists and organizations to bill for their HEP review and management activities, but despite CMS waivers during the COVID-19 pandemic, organizations may have the most success billing for these services if they are billed incident to and furnished under general supervision of a physician. Analysis of patient interaction in the system indicates relatively low adherence but offers a new avenue of objective data collection regarding adherence to HEP’s. If facilities are interested in investing in new systems of remote monitoring and treatment, we highly recommend designing a structured program for training and technical support throughout the initial implementation period. Though work needs to be done on identifying the optimal sensor information to report to therapists, therapists found the monitoring aspects of the system and the physical device to be motivating to their patients and reported increased adherence to prescribed HEP’s.

## Figures and Tables

**Table 1:** List of RPM codes and their descriptions. See Text S1 for further details.

Code	Description
99453	Initial set up and patient education
99454	Supply of devices and collection, transmission, and summary of services each 30 days
99457	First 20 min of remote physiologic monitoring by clinical staff, physician, or qualified healthcare provider in a calendar month
99458	For additional 20 min of remote physiologic monitoring by clinical staff, physician, or qualified healthcare provider in the same calendar month as 99457

**Table 2:** Survey results and time spent training by each therapist outside of training session delivered by study team.

Therapist	Self-Rating after Initial Training	Time Spent Training after Session I (h)	Time Spent Training after Session II (h)
Therapist 1	3	6	1
Therapist 2	3	1	1.5
Therapist 3	4	0	0.25

Self-ratings were reported in response to the question: “Now that the training session is complete, how comfortable do you feel using the SEAM system?” on a scale from 1 (very uncomfortable) to 5 (very comfortable).

**Table 3:** Participants’ (*n* = 9) payer sources, care setting, and care domain.

Patient ID	Payer Source	Care Setting	Care Domain (PT/OT)
SEAM 01	Aetna MC PPO	Outpatient	PT
SEAM 03	Medicare	Outpatient	OT
SEAM 04	Cigna PPO	Outpatient	OT
SEAM 05	Blue Cross	Outpatient	PT
SEAM 06	Blue Cross HMO	Inpatient	PT
SEAM 07	Blue Cross Blue Shield PPO	Outpatient	PT
SEAM 08	Blue Shield PPO	Outpatient	OT
SEAM 09	Healthy HMO	Outpatient	OT
SEAM 10	Blue Cross PPO	Outpatient	PT

**Table 4:** Patient prescriptions quantified as the average number of activities prescribed per day, the average number of sets prescribed per activity, the average number of repetitions prescribed per activity, the total number of days the system was in use, and the total number of activities prescribed over usage of the system. Therapists prescribed exercises to their patients every day except for SEAM 07 who was not prescribed exercises on Sundays.

Study ID	Average Activities per Day	Average Sets per Activity	Average Reps per Set	Total Number of Days	Total Number of Activities
SEAM 01	40	1	9	49	1973
SEAM 03	4	1	10	150	639
SEAM 04	14	1	10	16	218
SEAM 05	20	1	8	71	1437
SEAM 07	12	1	9	46	549
SEAM 08	11	1	10	135	1515
SEAM 09	12	1	12	116	1419
SEAM 10	11	1	7	74	823

SEAM 01 was prescribed an aerobic sequence of short exercises, giving them a higher average of activities per day than other patients. Patients were prescribed a broad variety of activities including strengthening, motor control, flexibility, and pain control activities.

**Table 5:** Adherence statistics per patient.

Study ID	Percent Active Days	Percent of Exercises Completed	Percent of Attempted Exercises with Puck Data
SEAM 01	24%	6%	35%
SEAM 03	24%	16%	0%
SEAM 04	6%	5%	0%
SEAM 05	54%	37%	76%
SEAM 07	35%	13%	36%
SEAM 08	52%	36%	16%
SEAM 09	34%	11%	0%
SEAM 10	92%	78%	0%

“Percent Active Days” indicates the percent of days, on which exercises were prescribed, that any level of activity was performed. “Percent of Exercises Completed” reports the percent of prescribed exercises that were completed in full, as determined by the Pt Pal software running through the full exercise sequence. “Percent of Exercises with Device Data” indicates the percent of prescribed exercises that were initiated for which data from the puck was recorded. Puck data was not collected for some exercises because of Bluetooth connectivity issues.

**Table 6:** Patient and therapist responses to questions at discharge. SEAM 04 self-discharged before the discharge survey could be delivered and thus the survey response only includes the parts from their therapist, Therapist 3.

Patient	Therapist	Questions and Answers
		Patient Question: What Did You Like Most about the System?
SEAM 01	Therapist 1	“It took me along progressively and I was able to watch the videos we recorded which helped.” Unfortunately, the patient accidentally deleted the videos.
SEAM 05	Therapist 2	“It kept me on task”
SEAM 07	Therapist 2	“I liked having the exercises organized by day and having the videos”
SEAM 04	Therapist 3	
		Patient Question: What did you like least about the system?
SEAM 01	Therapist 1	“It was hard and uncomfortable to put the puck on my arm.”
SEAM 05	Therapist 2	“The puck was a hassle, large and bulky and did not always capture the repetitions and hard to understand “
SEAM 07	Therapist 2	“The puck did not provide specific feedback so it didn’t seem beneficial”
SEAM 04	Therapist 3	
		Therapist Question: What did you find most useful about the SEAM system for treating this patient?
SEAM 01	Therapist 1	“The ability to record videos”
SEAM 05	Therapist 2	“Patient was motivated to use the system because they knew I was monitoring their completion of their exercises”
SEAM 07	Therapist 2	“Pt Pal”
SEAM 04	Therapist 3	“Being able to monitor compliance”
		Therapist Question: What was the most difficult or frustrating part of using the SEAM system with this patient?
SEAM 01	Therapist 1	“The multiple steps to use the system each time I saw the patient.”
SEAM 05	Therapist 2	“The puck was not useful for most of the exercises.”
SEAM 07	Therapist 2	“The puck was not very useful and the patient decided not to do Zoom after the 3rd visit and switched to in clinic visits for the remainder of the therapy”
SEAM 04	Therapist 3	“Time constraints for reviewing progress”

**Table 7:** Summary of content analysis results from end of study interviews with outpatient therapists.

Themes			Code: Descriptions or Examples	Number of Mentions by Therapist (T#)		
				T1	T2	T3
Positive	Value to Patients		adherence: compliance, adherence, accountability	0	2	4
			patient satisfaction: patients find the system interesting and engaging	3	2	2
			game mode: gamification aspects, mentions of engagement relative to gamified exercises	0	1	1
			patient motivation: motivation, excitement, having a physical device was motivating, motivation as a result of being monitored	4	1	1
			feedback to patients: system provides feedback to patients	1	0	0
	Facilitating Care		facilitate telerehab: communication with patients, remote monitoring	1	2	0
			billing: making non-productive time productive, difficulties related to billing	1	3	0
			order sets: convenience of creating order sets for prescribing	0	1	0
			adding exercises: ability to create custom exercises	0	1	0
			useful features: automatic Bluetooth connection, activity tracking, “copy to all exercises”	4	0	0
			monitoring: ability to monitor patient activity even when they are not in the clinic	1	1	3
			future and potential uses: to bridge gaps between evaluation and treatments	1	0	0
			feedback from patients: feedback allows modification of programs or preventing injury	0	0	2
Negative	Implementation Issues	Patient Selection	patient attributes: consistency in following instructions, diagnosis, cognition, fear of technology, preference for paper instructions, state their phone or data plan could not support the intervention	3	2	4
			patient unable to operate alone	4	0	0
			lack of caregiver support: patients have no one to assist them with the system in the home setting	2	0	0
		System Suitability	puck suitability for diagnoses: device does not capture data relevant to balance and stability exercises, patients have difficulty grasping puck	0	4	0
			game mode: exercises that therapists wanted were not available, suitability for diagnosis	2	1	0
		Training/ Education	survey: patients did not complete or selected answers straight down the middle, therapists were not trained how to optimally assign surveys	1	1	2
			billing: confusion, missed billing opportunities, improper sequence, no preauthorization	0	1	1
			exercise library: dissatisfaction with uniformity, not finding desired exercises, organization not what was expected, experience with other HEP software	2	1	0
			portal interface: difficulties navigating and using interface	2	0	1
	Potential System Improvements		reasons for non-complete: the reasons for non-complete did not line up with patients’ reasons	0	1	0
			number of steps: patient forgot app access code, Bluetooth workflow	5	0	0
	Technical Issues		app/software: software instability, number of steps required	1	2	0
			pucks not recording/performing as expected: reported counts are different than expected, difficulties connecting to Bluetooth	2	1	3
			Bluetooth problems: problems regarding making the Bluetooth connection	6	0	1
			facility internet	1	0	0
			patient frustration: patient frustration due to system not operating properly or as the patient believes it should	0	2	2

**Table 8:** List of reasons why specific RPM codes were denied by payers.

Payer	Code	Reason
Aetna Medicare Managed Care	99457	Non-Covered
	99458	Non-Covered
Medicare	99457	Non-Covered
	99458	Non-Covered
Cigna	99454	No Reason Given (one count of 99457 was reimbursed and one was not)
Blue Cross	99453	Contract for PT maxes at USD 317 per day
	99454	Contract for PT maxes at USD 317 per day
	99457	Contract for PT maxes at USD 317 per day
Blue Shield	99457	Non-Covered
	99458	Non-Covered
United Healthcare Medicare Managed Care	99457	Non-Covered (one count of 99457 was reimbursed and one was not)

**Table 9:** Estimated percent of eligible patient population that could benefit from the SEAM system, including descriptions of the general population and reasons they might not be eligible.

Therapist	Domain	%	General Patient Diagnoses	Reasons Patients Might Not Be Eligible
1	PT	22%	Movement disorders, neurologic conditions	Age, technological fluency, lack of smart phone, cognitive deficits from diagnosis
2	PT	75%	Lymphedema	Technological fluency, lack of smart phone, diagnosis related barriers
3	OT	100%	Neurologic conditions	

## Data Availability

Data agreements have not yet been made to facilitate potential use in a broader context.

## Supplementary Materials

The following are available online at https://www.mdpi.com/article/10.3390/ijerph181910186/s1, Text S1: Explanation of RPM codes as they might be billed for in relation to Pt Pal. This document was provided by Pt Pal for use during 2020, Text S2: Surveys used for data collection throughout the study (Post Training, Post Therapy Session, Patient Discharge), Text S3: Proposed SEAM Training Schedule, following the end of study interviews, the research team revised a proposed plan of training considering the training delivered during the study, common issues which occurred, and comments from the therapists during end of study interviews, Table S1: SEAM Study RPM Reimbursement Table, contains the full list of RPM codes billed and reimbursed during the 2020 billing cycle for the study. Billing details are shown by patient, and any reasons for denied codes are listed.
