# Supplementary material for: A Pilot Study of a Sensor Enhanced Activity Management System for Promoting Home Rehabilitation Exercise Performed during the COVID-19 Pandemic: Therapist Experience, Reimbursement, and Recommendations for Implementation
Source: Int J Environ Res Public Health. 2021 Sep 28;18(19):10186. doi: 10.3390/ijerph181910186 (PMC8508164; doi:10.3390/ijerph181910186)
Supplement: Supplementary file 1 [file ijerph-18-10186-s001.zip › Table S1 - SEAM Study RPM Reimbursement Table.pdf]

SEAM ID	Days of Service	Department	Payer Name	CPT	Charge	Reimb	CPT	Charge	Reimb	Notes
1	30	PT	AETNA MEDICARE MANAGED CARE	99453	$ 140.00	$ 134.42	99454	$ 42.03		99457 & 99458 Non-Covered
	31									Denied
3	28	OT	MEDICARE	99453	$ 140.00	$ 134.42	99454	$ 47.00	$ -	99454 pmt included in allowance for 99453
	28			99457 x 3	$ 139.00		99458	$ 139.00		Non-Covered
	31			99454	$ 47.00	$ 42.03				99457 & 99458 Non-Covered
	28			99457	$ 139.00					Non-Covered
4	30	OT	CIGNA	99453	$ 140.00	$ 134.68	99454	$ 45.21		
	30			99457	$ 139.00	$ 133.72	99457	$ 133.72		
7	28	PT	BLUE CROSS	99457	$ 139.00					Contract for PT maxes at $317 per day
	31			99457	$ 139.00					Contract for PT maxes at $317 per day
8	31	OT	BLUE SHIELD	99457	$ 139.00					non-novered
	29			99458	$ 90.00					Non-Covered
9	25	OT	UNITED HEALTHCARE MEDICARE MANAGED CARE	99457	$ 139.00					non-covered
	26			99458	$ 90.00	$ 34.97	99457	$ 139.00	$ 34.97	
10	23	PT	BLUE CROSS	99453	$ 140.00		99454	$ 47.00		Contract for PT maxes at $317 per day
