# Supplementary material for: A Pilot Study of a Sensor Enhanced Activity Management System for Promoting Home Rehabilitation Exercise Performed during the COVID-19 Pandemic: Therapist Experience, Reimbursement, and Recommendations for Implementation
Source: Int J Environ Res Public Health. 2021 Sep 28;18(19):10186. doi: 10.3390/ijerph181910186 (PMC8508164; doi:10.3390/ijerph181910186)
Supplement: Supplementary file 1 [file ijerph-18-10186-s001.zip › Text S1 2020 Pt Pal Billing 30 Patients.pdf]

Billing for Pt Pal

Chronic Care Remote Physiologic Monitoring (CPT Codes 99453, 99454, 99457 and 99458)

- **CPT code 99453** (Remote monitoring of physiologic parameter(s) (eg, weight, blood pressure, pulse oximetry, respiratory flow rate), initial; set-up and patient education on use of equipment) about $20

- **CPT code 99454** (Remote monitoring of physiologic parameter(s) (eg, weight, blood pressure, pulse oximetry, respiratory flow rate), initial; device(s) supply with daily recording(s) or programmed alert(s) transmission, each 30 days) about $63

- **CPT code 99457** (Remote physiologic monitoring treatment management services, 20 minutes or more of clinical staff/physician/other qualified healthcare professional time in a calendar month requiring interactive communication with the patient/caregiver during the month) about $51

- **CPT code 99458** is to reimburse for subsequent 20 minute intervals of RPM services provided by clinical staff, physician, or QHCP spent above and beyond the initial 20 minutes in a calendar month indicated for CPT Code 99457 for about $42

Some notable improvements from previous rules include:

- Reimbursement for the time spent preparing devices & health technology for patients and training patients on how to use the technology, applications and devices.

- CPT Code 99457 allows services to be performed by "clinical staff" as well as physicians and other qualified health care professionals. The term "clinical staff" was not included in 2018 unbundled CPT code 99091 (which was 16 years old). Clinical staff includes, for example, PTs, OTs, SLPs, RNs and Medical Assistants. All practitioners must practice in accordance with applicable state law and scope of practice laws.

- CPT code 99457 and 99458 are billed based on a calendar month, not a 30 day period. This is much easier for tracking and claims submissions.

- Compared with the 30 minutes of time required for CPT 99091, CPT 99457 only requires 20 minutes of interactive communication with patient/caregiver.

The patient must consent to participating in remote patient monitoring services, and if the patient is new, or has not been into the clinic in over a year, a face-to-face visit is required, for example, for an annual wellness visit.

Three codes were introduced in January 2019 that support the implementation, provisioning, and use of remote physiological monitoring technology for patients. CPT code 99457 referred to the 20 minutes per month of clinical professional treatment management time, and when first released, the services were only allowed to be furnished by the physician / practitioner, not auxiliary personnel "incident to" a physician. This was not an ideal scenario, as many remote monitoring programs are managed by staff that report to a physician, and patients routinely monitored by nurses, and other healthcare professionals, that can triage to a physician as needed.

The technical correction stated that CPT code 99457 CAN be billed "incident to" by licensed medical professionals, under direct supervision (in the same building) of a physician. This correction allows care staff, such as therapists and nurses, to monitor patients and review data, enabling physicians to focus their efforts on patients that need the most attention. This clarification improves the scalability and workflows of remote monitoring programs and will enable more patients to receive enhanced care through remote monitoring.

Potential Revenue

In an example scenario where 30 patients were enrolled per month (360 annually), the facility would be able to charge:

1. the initial, one-time enrollment charge (99453) of $20 x 30 = $600
2. data capture device (Pt Pal) (99454) for $63 x 30 = $1,890
3. reviewing the results (99457) from the patient for $51 x 30 = $1,530
4. each additional 20 minute block (99458) spent reviewing patient-generated health data within the 30-day window (3 charges of $42 = $126 x 30 = $3,780)

- Total charges for 30 patients in Month 1 = $7,770

In an example scenario, if the staff enrolled a patient in Pt Pal, logged 20 minutes of time reviewing data in Week 1, then logged 20 additional minutes per week in Week 2, Week 3 and Week 4, the total charges in the first 30-day window would be $259.

In Month 2 (M2), if you enrolled another 30 patients, you would again bill $600 for patient enrollment (99453), and now would charge for data capture device (99454) and results review (99457) for 60 active patients (M1 30 + M2 30 = 60), totaling $35,670 for the month. If you add another 60 in Month 3, and then maintain 240 active

patients, your charges would be $29,400 per month ($7,350 p/week). For planning purposes, spending 80 minutes per month monitoring 120 active patients requires 40 hours of staff time per week.

Example Scenario

In this example scenario, we are estimating using 30 new patients per month for an average 4-month active cycle.

Thirty Patients per month for 12 months

(anticipating 30 patients per month dropping off after 4 months)

# of Patients	Jan / 30 pts	Feb / 60 pts	Mar / 90 pts	Apr / 120 pts	May / 120 pts	June / 120 pts	July / 120 pts	Aug / 120 pts	Sept / 120 pts	Oct / 120 pts	Nov / 120 pts	Dec / 120 pts
**CPT code 99453 ($20)**	$600	$600	$600	$600	$600	$600	$600	$600	$600	$600	$600	$600
**CPT code 99454 ($63)**	$1,890	$3,780	$5,670	$7,560	$7,560	$7,560	$7,560	$7,560	$7,560	$7,560	$7,560	$7,560
**CPT code 99457 ($51)**	$1,530	$3,060	$4,590	$6,120	$6,120	$6,120	$6,120	$6,120	$6,120	$6,120	$6,120	$6,120
**CPT code 99458 ($42)*3 p/30 days**	$3,780	$7,560	$11,340	$15,120	$15,120	$15,120	$15,120	$15,120	$15,120	$15,120	$15,120	$15,120
Monthly Charges	$7,800	$15,000	$22,200	$29,400	$29,400	$29,400	$29,400	$29,400	$29,400	$29,400	$29,400	$29,400
Total Charges p/year:				$309,600								
