# Supplementary material for: A Pilot Study of a Sensor Enhanced Activity Management System for Promoting Home Rehabilitation Exercise Performed during the COVID-19 Pandemic: Therapist Experience, Reimbursement, and Recommendations for Implementation
Source: Int J Environ Res Public Health. 2021 Sep 28;18(19):10186. doi: 10.3390/ijerph181910186 (PMC8508164; doi:10.3390/ijerph181910186)
Supplement: Supplementary file 1 [file ijerph-18-10186-s001.zip › Text S2 - Data Collection Surveys.pdf]

Post Training Survey

Please provide some feedback on today's training session. This will help us tailor our follow up training session.

1. Name

   ____

2. Now that the training session is complete, how comfortable do you feel using the SEAM system?

   Mark only one oval.

	1	2	3	4	5	
Very Uncomfortable	◯	◯	◯	◯	◯	Very Comfortable

3. Which component do you think is most useful?

   Mark only one oval.

   ◯ Pt Pal: portal to assign exercises and collect information
   ◯ Zoom: video conference tool to remotely communicate with patients
   ◯ Rep Mode: uses Fitmi pucks to collect repetition data
   ◯ Game Mode: uses FitMi pucks to collect repetition data from gamified exercises

4. Which component do you think is most confusing?

   Mark only one oval.

   ◯ Pt Pal: portal to assign exercises and collect information
   ◯ Zoom: video conference tool to remotely communicate with patients
   ◯ Rep Mode: uses Fitmi pucks to collect repetition data
   ◯ Game Mode: uses FitMi pucks to collect repetition data from gamified exercises

5. What part of the process is most difficult for you?

6. What part of the process do you think will be most difficult for patients?

7. What would you like more help with during our follow up training?

This content is neither created nor endorsed by Google.

Google Forms

Post Therapy Session Survey

Please fill this survey out after every session with a patient in this study. Remember that we cannot collect any personally identifying information about the patients, so please refer to them using the ID's from the Study Participant ID's sheet.

At anytime, you may also record notes in the Study Observation Notes document.

1. Please enter your name:

____

2. What is the study ID of the patient you saw today?

____

3. What features of SEAM did you use with this patient today? (select all that apply)

Check all that apply.

- [ ] Pt Pal
- [ ] Zoom
- [ ] Rep Mode
- [ ] Game Mode
- [ ] Puck Mat
- [ ] Puck Strap

4. How easy or difficult was it for you to use SEAM with this patient today?

Mark only one oval.

	1	2	3	4	5	
Very Difficult	◯	◯	◯	◯	◯	Very Easy

5. What went well with using SEAM today?

6. What did not go well with using SEAM today?

7. How satisfied or dissatisfied does your patient appear to be with the SEAM system?

Mark only one oval.

	1	2	3	4	5	
Very Dissatisfied	⬭	⬭	⬭	⬭	⬭	Very Satisfied

8. What insurance provider does this patient use?

9. How easy or difficult was it for you to bill the patient's insurance for time spent using SEAM?

Mark only one oval.

	1	2	3	4	5	
Very Easy	⬭	⬭	⬭	⬭	⬭	Very Difficult

10. What billing code did you use? (enter as many as necessary separated by a space)

11. Other notes about this session:

This content is neither created nor endorsed by Google.

Google Forms

Patient Discharge Survey

Please fill this survey out with the patient when they are discharged from your care. Remember that we cannot collect any personally identifying information about the patients, so please refer to them using the ID's from the Study Participant ID's sheet.

At anytime, you may also record notes in the Study Observation Notes document.

1. Please enter your name:

____

2. What is the study ID of the patient?

____

For the patient

Please fill this out with responses from your patient about using the SEAM system.

3. I think that doing this activity was useful for my physical therapy.

Mark only one oval.

	1	2	3	4	5	6	7	
Not at all true	◯	◯	◯	◯	◯	◯	◯	Very true

4. This activity did not hold my attention at all.

Mark only one oval.

	1	2	3	4	5	6	7	
Not at all true	◯	◯	◯	◯	◯	◯	◯	Very true

5. It was important to me to do well at this task.

Mark only one oval.

	1	2	3	4	5	6	7	
Not at all true	◯	◯	◯	◯	◯	◯	◯	Very true

6. This was an activity that I couldn't do very well.

Mark only one oval.

	1	2	3	4	5	6	7	
Not at all true	◯	◯	◯	◯	◯	◯	◯	Very true

7. I thought this activity was quite enjoyable.

Mark only one oval.

	1	2	3	4	5	6	7	
Not at all true	◯	◯	◯	◯	◯	◯	◯	Very true

8. I put a lot of effort into this.

Mark only one oval.

	1	2	3	4	5	6	7	
Not at all true	◯	◯	◯	◯	◯	◯	◯	Very true

9. After working at this activity for a while, I felt pretty competent.

Mark only one oval.

	1	2	3	4	5	6	7	
Not at all true	◯	◯	◯	◯	◯	◯	◯	Very true

10. I believe doing this activity could be beneficial to me.

Mark only one oval.

	1	2	3	4	5	6	7	
Not at all true	◯	◯	◯	◯	◯	◯	◯	Very true

11. What did you like most about the system?

____
____
____
____
____

12. What did you like least about the system?

____
____
____
____
____

13. What was the most difficult part of using the system?

14. How likely are you to continue using the system after you are discharged?

Mark only one oval.

- Very Likely
- Likely
- Not Sure
- Unlikely
- Very Unlikely

If you performed telerehab with this patient, please fill out the following questions.

15. Do you agree or disagree with this statement: This system made it easier to perform telerehab.

Mark only one oval.

- Strongly Agree
- Agree
- Neutral
- Disagree
- Strongly Disagree

16. Do you agree or disagree with this statement: Using this system made me more comfortable about getting physical therapy during the COVID19 pandemic.

Mark only one oval.

- Strongly Agree
- Agree
- Neutral
- Disagree
- Strongly Disagree

For the therapist

Please fill this out according to your experience with this patient

17. Which features of SEAM did you use with this patient? (select all that apply)

Check all that apply.

- [ ] Pt Pal
- [ ] Zoom
- [ ] Rep Mode
- [ ] Game Mode
- [ ] Puck Strap

Other: [ ] ____________________

18. How easy or difficult was it for you to use SEAM with this patient overall?

Mark only one oval.

	1	2	3	4	5	
Very Difficult	○	○	○	○	○	Very Easy

19. What did you find most useful about the SEAM system for treating this patient?

20. What was the most difficult or frustrating part of using the SEAM system with this patient?

21. How satisfied or dissatisfied does your patient appear to be with the SEAM system overall?

Mark only one oval.

	1	2	3	4	5	
Very Dissatisfied	◯	◯	◯	◯	◯	Very Satisfied

22. How easy or difficult was it for you to bill the patient's insurance for time spent using SEAM?

Mark only one oval.

	1	2	3	4	5	
Very Easy	◯	◯	◯	◯	◯	Very Difficult

23. Other notes about this patient:

This content is neither created nor endorsed by Google.

Google Forms
