# Supplementary material for: A Pilot Study of a Sensor Enhanced Activity Management System for Promoting Home Rehabilitation Exercise Performed during the COVID-19 Pandemic: Therapist Experience, Reimbursement, and Recommendations for Implementation
Source: Int J Environ Res Public Health. 2021 Sep 28;18(19):10186. doi: 10.3390/ijerph181910186 (PMC8508164; doi:10.3390/ijerph181910186)
Supplement: Supplementary file 1 [file ijerph-18-10186-s001.zip › Text S3 - Proposed SEAM Training Schedule.pdf]

Proposed SEAM Training Schedule

Pre-training: survey on patient population/desired exercises

Initial Training (8 hours)

1. Morning
   - Background/ Aims of the study
   - Pt Pal introduction and navigations
   - Puck/ Clip introduction
   - Android/ iPhone manuals
   - Billing information
   - Documentation in Epic
2. Afternoon
   - Create account
   - Download app on phones
   - Create “testing subjects”
   - Develop sample order set
   - Prescribe exercise program
   - Perform exercises using app on phone and puck/clip
   - Adjust exercises from the Pt Pal portal
   - Refresh to make sure “subjects” receive updated exercise program

Follow up Training Meeting (4 hours)

- Q&A
- Quiz on “test subject” using different case scenarios: adding exercises, modifying exercise prescription, obtaining reports from Pt Pal, submitting appropriate billing codes, etc.

Weekly or bi-weekly meetings (1 hour)

- Enrollment updates
- Troubleshooting
- Hardware/software updates
- Billing reminder

Documentation/ Data or progress retrieval

- In Epic – patient’s chart
- Pt Pal

Technical support hotline

- Monitor seam system usage
- Monitor billing submission
